# Spontaneous ilio-psoas hematomas complicating intensive care unit hospitalizations

**DOI:** 10.1371/journal.pone.0211680

**Published:** 2019-02-22

**Authors:** Thierry Artzner, Raphaël Clere-Jehl, Malika Schenck, Michel Greget, Hamid Merdji, Pierre De Marini, Nicolas Tuzin, Julie Helms, Ferhat Meziani

**Affiliations:** 1 Université de Strasbourg (UNISTRA), Faculté de Médecine, Hôpitaux universitaires de Strasbourg, Service de Réanimation Médicale, Nouvel Hôpital Civil, Strasbourg, France; 2 ImmunoRhumatologie Moléculaire, INSERM UMR_S1109, LabEx TRANSPLANTEX, Centre de Recherche d’Immunologie et d’Hématologie, Faculté de Médecine, Fédération Hospitalo-Universitaire (FHU) OMICARE, Fédération de Médecine Translationnelle de Strasbourg (FMTS), Université de Strasbourg, Strasbourg, France; 3 Service de Réanimation Médicale, Hôpital de Hautepierre, Hôpitaux Universitaires de Strasbourg, Strasbourg, France; 4 Service d’Imagerie Interventionnelle, Hôpitaux Universitaires de Strasbourg, France; 5 INSERM (French National Institute of Health and Medical Research), UMR 1260, Regenerative Nanomedicine (RNM), FMTS, Strasbourg, France; 6 Laboratoire de Biostatistique et d’Informatique Médicale, ICube UMR 7357, Faculté de Médecine, Hôpitaux Universitaires de Strasbourg, Strasbourg, France; 7 Groupe Méthode en Recherche Clinique, Service de Santé Publique, Hôpitaux Universitaires de Strasbourg, Strasbourg, France; Azienda Ospedaliero Universitaria Careggi, ITALY

## Abstract

**Background:**

Ilio-psoas hematoma is a potentially lethal condition that can arise during hospital stay. However, neither the incidence nor the prognosis of patients whose stay in intensive care units (ICU) is complicated by a iatrogenic ilio-psoas hematoma is known.

**Methods:**

A bicentric retrospective study was conducted to compile the patients who developed an ilio-psoas hematoma while they were hospitalized in ICU between January 2009 and December 2016. Their biometric characteristics, pre-existing conditions, the circumstances in which the hematoma was diagnosed, the treatments they received and their prognosis were recorded.

**Results:**

Forty patients were diagnosed with an ilio-psoas hematoma during their ICU stay. The incidence of this complication was 3.8 cases for 1000 admissions, taking into account only patients who stayed more than three days in ICU. The median age of patients was 74 years old and the median time between admission and the diagnosis of ilio-psoas hematoma was 12.6 days. A large proportion of them was obese (42.5%) and/or under dialysis (50%) prior to developing their hematoma. Ninety-five percent of the patients had heparin at prophylactic or therapeutic doses. Only 10% of them were above the therapeutic range of anticoagulation. The ICU mortality rate was of 50% following this complication (versus a general mortality rate of 22% for the patients without IPH over the same period of time). Patients with IPH that were complicated by disseminated intravascular coagulopathy had a significantly higher mortality rate than those with IPH and no disseminated intravascular coagulopathy (OR 6.91, 95% CI [1.28; 58.8], *p* = 0.04).

**Conclusion:**

Age, anticoagulation, a high body mass index and dialysis seem to be risk factors of developing an ilio-psoas hematoma in ICU. Iatrogenic ilio-psoas hematomas complicated by disseminated intravascular coagulopathies are more at risk of leading to death. It is noteworthy that activated partial thromboplastin time above the therapeutic range was not a good predictor of developing a hematoma for patients who received unfractioned heparin therapy.

## Background

The management of thrombotic and hemorrhagic risk in intensive care unit (ICU) is a complex and constant issue for clinicians. Ilio-psoas hematomas (IPH) may stand among the most serious hemorrhagic adverse effects complicating an ICU stay. However, the incidence of IPH among patients hospitalized in ICU remains unknown and its effect on patient outcome has classically not been reported in studies of mortality factors impacting ICU patients [1, 2]. Early identification of patients at risk of developing IPH during their ICU stay may allow early diagnosis and treatment to decrease IPH-imputed mortality.

While some risk factors of non-traumatic IPH (anticoagulation therapy, old age and hemodialysis) have been identified [3], the pathophysiology of this condition is still unclear. Furthermore, although well-documented, its clinical presentation outside ICU is unspecific [4,5,6] it includes abdominal, back or groin pain, anemia, positive psoas sign, the presence of an iliac mass, femoral neuropathy or hemodynamic instability. Clinicians therefore usually need further exploration to diagnose IPH or to confirm a clinical suspicion of IPH. While ultrasonography used to be the preferred mode of diagnosis of IPH [7], it has now been largely supplanted by computerized tomography [3].

The prognosis of IPH ranges from benignity to rapid lethality, due to hemorrhagic shock. To date, there are no guidelines devoted to the treatment of IPH, in particular concerning the alternative between conservative treatment and the use of either embolization or surgery. A review has shown that benign cases tend to be treated conservatively, while embolization techniques are used for more severe cases and surgery is only used as a last resort option in the most severe cases [3].

ICU patients are at risk of developing both hemorrhagic and thrombotic complications that may significantly burden their stay and alter their prognosis. They often combine one or more risk factors of IPH during their stay: old age, anticoagulation therapy and hemodialysis [3]. To our knowledge, there is no published work specifically studying the incidence of IPH among patients while they are hospitalized in ICU and the effect this condition has on their stay. In this retrospective bicentric study, we determine the incidence of IPH in ICU, we identify potential risk factors and describe their treatment and outcome.

## Methods

### Patients

Patients who developed an IPH while they were hospitalized in one of the two medical ICU (55 beds in total) from Strasbourg university hospitals (France) were included. The study period ran from January 2009 to December 2016. In order to ensure that we were only studying patients who developed IPH as a complication of their ICU stay, we excluded the patients who were admitted for or with IPH or whose IPH was diagnosed within the first 72 hours of their stay.

### Data collection

All the patients who were admitted in the ICU and stayed there for more than 72 hours from January 2009 to December 2016 were screened for inclusion in the study by searching for the following keywords in their digital medical charts: “iliac”, “psoas”, “ilio-psoas”, “retroperitoneal’ and “hematoma”.

Patients’ biometric characteristics and pre-existing conditions, along with their initial diagnosis and prognosis using the Simplified acute gravity score II (SAPS II) were recorded. The treatments that patients received during their hospital stay prior to the diagnosis of IPH, which may have contributed to the hemorrhagic event, were recorded: antiplatelet and anticoagulant therapy, invasive arterial procedures such as extracorporeal membrane oxygenation (ECMO), coronarography, insertion of transcatheter aortic valve implantation (TAVI) and intra aortic balloon counter pulsation (IABCP).

The clinical circumstances in which the diagnosis of IPH was made were recorded, as well as potential signs of active bleeding on the CT scan. The time of diagnosis was defined as the time at which the CT scan showing the IPH was performed.

Patients’ biological results were recorded over the period ranging from 48 hours prior to the diagnosis to 48 hours after. Over-anticoagulation was defined as an activated partial thromboplastin time (APTT) ratio > 3 at any point during the 48 hours preceding the diagnosis of IPH for patients who received unfractionated heparin. Over-anticoagulation for patients with vitamin K antagonist (VKA) treatment was defined as an international normalized ratio (INR) > 3 and INR > 3.5 for patients with a prosthetic mechanical heart valve at any point during the 48 hours preceding the diagnosis of IPH. To determine whether patients developed a disseminated intravascular coagulopathy (DIC), we used the Japanese association for acute medicine (JAAM) criteria [8].

Hemorrhagic shock was defined according to international criteria as hemodynamic instability defined by systolic arterial blood pressure (SAP) < 90 mmHg or signs of shock and the presence of a life-threatening bleeding or bleeding that compromises vital function [9].

IPH treatment and prognosis was recorded: blood products transfusion, the volume of fluid replacement, the introduction or increase of vasopressor therapy (defined by a 20% increase during the 24 hours surrounding the diagnosis of IPH), the use of antifibrinolytic or prothrombotic agents (tranexamic acid, fibrinogen replacement therapy, vitamin K, prothrombin complex concentrates) and the use of protamine reversal. The use of surgical procedure or embolization (and the delay between diagnosis and embolization) was also recorded.

Therapeutic decisions were taken according to physician judgment and after pluridisciplinary discussion involving intensivists, surgeons and radiologists. Surgery and embolization were available around the clock at both centers.

### Statistical analysis

Quantitative parameters and continuous data are presented as median and interquartile range (IQR, 25th–75th percentile). Qualitative parameters are presented as counts and percentage. Categorical variables were compared using the Chi-square test or Fischer’s exact test, as appropriate. A multivariate analysis was performed to identify independent factors using a binomial logistic regression. Variables included in the multivariate analysis were those significant (*p* < 0.06) in univariate analysis (a Student test was used for quantitative variables following a normal distribution and a Mann-Whitney test was used for other variables), and we calculated the odds ratio (OR) associated and its 95% confidence interval (CI). All statistical analyses were carried out using R Core Team (2015) software.

The study design was approved by the Strasbourg university hospital ethics committee (ref 2017–43).

## Results

### IPH is a rare complication of ICU stay

**Fig 1:**
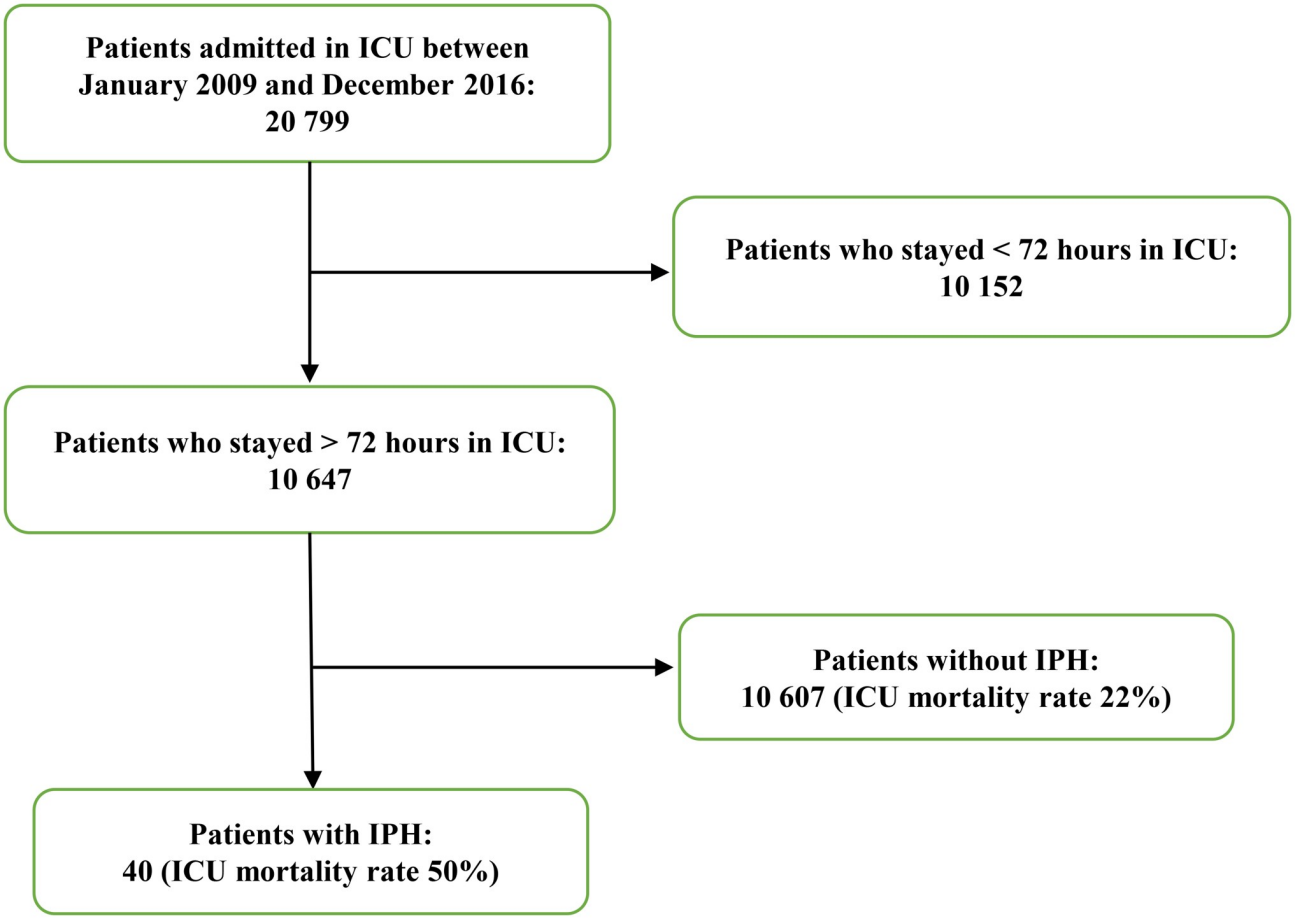
Study flowchart.

Between January 2009 and December 2016, 10 647 patients were hospitalized for more than 72 hours in the ICU we studied (see Fig 1). Forty patients developed IPH during their stay. This corresponds to 3.8 cases for 1000 admissions during the study period.

### Characteristics of patients developing IPH in ICU: High prevalence of old age and obesity

Median age on admission was 74 years and 16 (40%) patients were women (see Table 1). The median SAPS II score on admission was 58. The median Body Mass Index (BMI) was 27kg/m^2^. It is noteworthy that seventeen (42.5%) patients had a BMI above 30 and six (15%) had a BMI above 40. A significant proportion of the patients in the cohort had cardiovascular risk factors: hypertension (55%), chronic cardiac failure (45%), smoking (37.5%), type 2 diabetes (37.5%).

**Table 1:** Demographics, pre-existing conditions and reason for admission of patients.

**Demographics**	
Age, *years*	74 (66, 79)
Female gender, *n (%)*	16 (40)
Simplified Acute Gravity Score II (SAPS II)	58 (52–70)
**Pre-existing conditions**	
Body Mass Index, *kg/m*^*2*^	27 (23–32)
Obesity (Body Mass Index > 30 kg/m^2^), *n (%)*	17 (42.5)
Morbid obesity (Body Mass Index > 40 kg/m^2^), *n (%)*	6 (15)
Hypertension *n (%)*	22 (55)
Chronic cardiac failure, *n (%)*	18 (45)
Smoking *n (%)*	15 (37.5)
Type 2 diabetes *n (%)*	15 (37.5)
Peripheral artery disease, *n (%)*	8 (20)
Stroke, *n (%)*	4 (10)
Atrial Fibrillation, *n (%)*	14 (35)
Prosthetic mechanical heart valve, *n (%)*	2 (5)
Chronic kidney failure, *n (%)*	11 (27.5)
Chronic dialysis, *n (%)*	2 (5)
Type 1 diabetes *n (%)*	0
Cancer or hematological malignancy, *n (%)*	9 (22.5)
Remission, *n (%)*	4 (10)
Evolutive, *n (%)*	5 (12.5)
Chronic obstructive pulmonary disease	7 (17.5)
Organ transplant	4 (10)
Chronic alcoholism	5 (12.5)
Cirrhosis	1 (2.5)
**Reason for admission**	
Septic shock, *n (%)*	12 (30)
Cardiogenic shock, *n (%)*	11 (27.5)
Hemorrhagic shock, *n (%)*	2 (5)
Cardiac arrest, *n (%)*	3 (7.5)
Myocardial infarction, *n (%)*	4 (10)
Acute respiratory failure, *n (%)*	16 (40)
Coma, *n (%)*	2 (5)

Quantitative parameters are presented as median and interquartile range (IGR, 25^th^-75^th^ percentile).

### IPH diagnosis circumstances: Loss of red blood cells and shock are the most frequent signs leading to the diagnosis of IPH occurring in ICU

**Fig 2:**
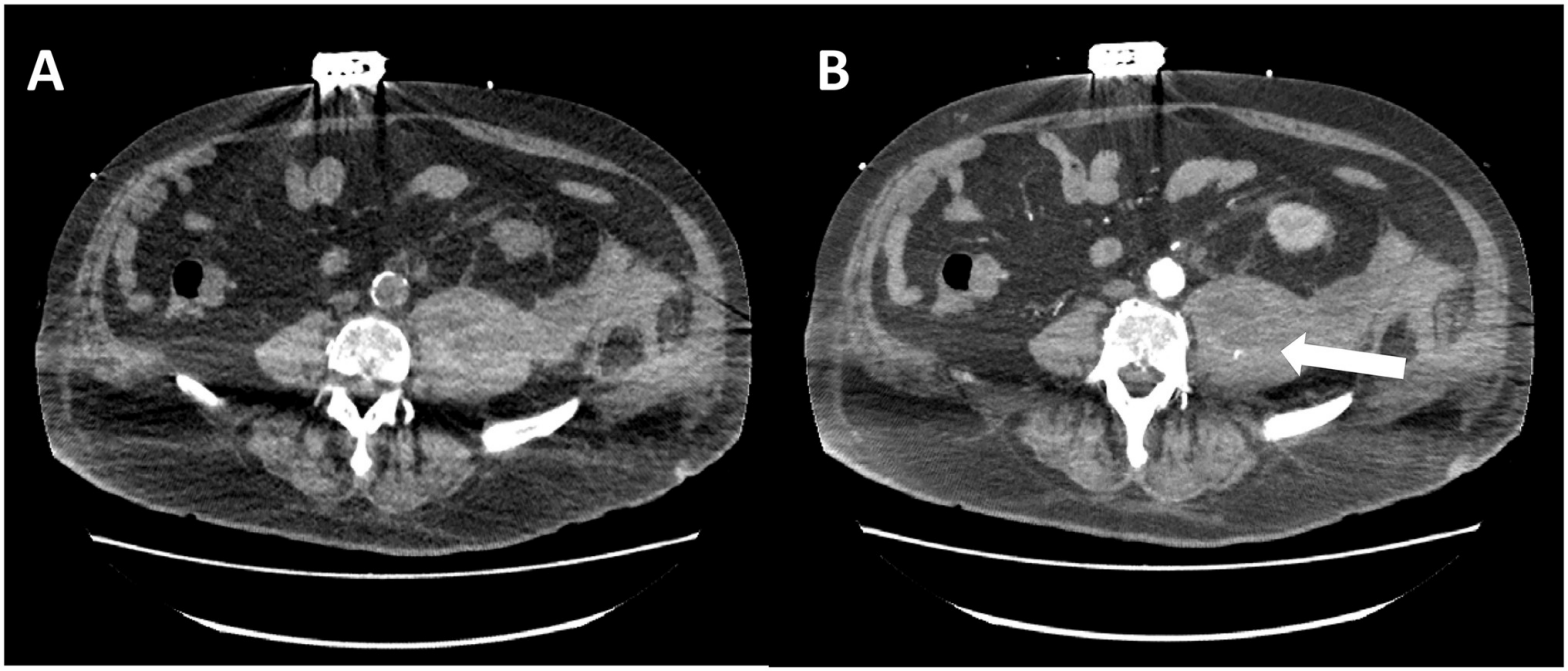
Spontaneous left psoas hematoma in a 70-year-old patient. **(A) Axial CT scan of the abdomen without contrast shows swelling of the left psoas with retroperitoneal diffusion (B) Axial contrast-enhanced CT scan shows extravasation of contrast medium (arrow)**.

The median time between admission and diagnosis was 12.6 days (see Table 2). The most frequent sign leading clinicians to order a CT scan was a loss of red blood cells of at least 2 g/dl present in 30 (75%) patients, followed by the apparition of shock or an aggravation of shock in 20 (50%) patients and pain or abdominal guarding in 12 (30%) patients. The diagnosis was made fortuitously in 2 (5%) cases and 3 (7.5%) patients suffered from cardiac arrest while they were hospitalized in the ICU immediately before the diagnosis of IPH was made. The median Sequential Organ Failure Assessments (SOFA) score on the day of diagnosis was 9 and the CT scan that helped diagnose the IPH revealed an active bleeding in 24 cases (60%), as illustrated in Fig 2.

**Table 2:** Diagnostic circumstances of IPH.

Time between admission and diagnosis, *days*	12.6 (0–36)
Loss of red blood cells, *n (%)*	30 (75)
Shock, *n (%)*	20 (50)
Pain, *n (%)*	12 (30)
Superficial hematoma, *n (%)*	3 (7.5)
Positive psoas sign, *n (%)*	2 (5)
Cardiac arrest, *n (%)*	3 (5)
Fortuitous, *n (%)*	2 (5)
Sequential Organ Failure Assessments (SOFA) score on day of diagnosis	9 (8–12)
Active bleeding on CT scan, *n (%)*	24 (60)

Quantitative parameters are presented as median and interquartile range (IGR, 25^th^-75^th^ percentile).

### Antiplatelet and anticoagulant therapy, as well as dialysis, are associated with IPH

Twenty (50%) patients were on dialysis while they were hospitalized at the ICU and prior to developing an IPH (see Table 3). None of these patients were on chronic dialysis beforehand. Fifteen (37.5%) patients had one or two antiplatelet drugs. Two (5%) patients did not have any anticoagulant drugs, 16 (40%) patients had prophylactic doses of heparin and 22 (55%) had therapeutic doses of heparin (14 patients for atrial fibrillation, 3 for venous thromboembolism disease, 2 for mechanical heart valves, 1 for acute coronary syndrome and 2 patients had therapeutic doses with no indication found in their medical charts). Unfractionated heparin was the anticoagulant drug used for all the patients in this study (no patient was treated with low molecular weight heparin or novel oral anticoagulant while they were in ICU). Two (5%) patients were undergoing a VKA-heparin overlap when the IPH occurred. In the 48 hours preceding the diagnosis of IPH, only 4 (10%) patients had APTT ratios above the therapeutic range (all the patients had an APTT done at least daily in the 48 hours prior to the diagnosis of IPH). The two patients who were undergoing a VKA-heparin overlap had APTT ratios and INR within therapeutic range. Antiplatelet therapy was not significantly associated with higher mortality rate in our cohort of patients with IPH. Thrombocytopenia was not significantly associated with mortality either.

**Table 3:** Treatment in ICU prior to IPH diagnosis.

Dialysis, *n (%)*	20 (50)
Antiplatelet therapy, *n (%)*	9 (22.5)
Double antiplatelet therapy, *n (%)*	6 (15)
Therapeutic anticoagulation, *n (%)*	22 (55)
Prophylactic anticoagulation, *n (%)*	16 (40)
Activated partial thromboplastin time above therapeutic range, *n (%)*	4 (10)
No anticoagulation, *n (%)*	2 (5)
Vitamine K antagonist (VKA)-heparin overlap, *n (%)*	2 (5)
ExtraCorporeal Membrane Oxygenation (ECMO), *n (%)*	3 (7.5)
Coronarography, *n (%)*	5 (12.5)
Intra Aortic Balloon Counter Pulsation (IABCP), *n (%)*	2 (5)
Transcatheter Aortic Valve Implantation (TAVI), *n (%)*	1 (2.5)
Thrombolysis, *n (%)*	0

Eleven (27.5%) patients underwent an invasive arterial procedure (veno-arterial ECMO, coronarography, IABPC or TAVI) in the days prior to developing IPH.

### More than half of the patients in our cohort were treated with embolization. Few received a anticoagulation reversal treatment

Twenty-one (52.5%) patients underwent an embolization procedure (see Table 4). The median delay between the diagnosis and the embolization procedure was 223 minutes. Four types of agents were used: permanent embolization microspheres (9 patients), resorbable embolization particles (11 patients), coils (3 patients) and liquid embolic agents (2 patients). In 7 cases, a combination of techniques was used (the most common was permanent embolization microspheres with resorbable embolization particles). The size of these samples did not allow to show a pattern between the type of procedure used and the outcome.

**Table 4:** Treatment and prognosis of patients developing IPH in the ICU.

**Treatment**	
Embolization, *n (%)*	21 (52.5)
Delay between diagnosis and embolization, *minutes*	223 (116, 376)
Surgery, *n (%)*	2 (5)
Introduction of a vasopressor drug, *n (%)*	23 (57.5)
Increase of a vasopressor drug, *n (%)*	7 (17.5)
Maximum dose of Norepinephrine in the 24 hours following diagnosis, *μg/kg/min*	0.38 (0.2–1.55)
Red cell concentrates, *units*	7 (4–8)
Plasma transfusion, *units*	2 (0–4)
Platelet transfusion, *units*	0 (0–1)
Volume of fluid challenge, *ml*	500 (0–1125)
Fibrinogen replacement therapy, *n (%)*	1 (2.5)
Vitamin K, *n (%)*	0
Protamine reversal, *n (%)*	4 (10)
Tranexamic acid, *n (%)*	1 (2.5)
Prothrombin complex concentrates, *n (%)*	1 (2.5)
**Prognosis**	
Duration of hospital stay, *days*	20.5 (16, 38)
Duration of mechanical ventilation, *days*	15 (10–17)
Reintubation, *n (%)*	10 (25)
Disseminated intravascular coagulopathy JAAM score	2 (2–4)
Disseminated intravascular coagulopathy, *n (%)*	11 (27.5)
Minimum platelet counts in the 48 hours following the diagnosis of IPH, *n/mm*^*3*^	135 000 (77 000–189 000)
Thrombocytopenia, *n (%)*	26 (65)
Maximum lactatemia in the 48 hours following the diagnosis of IPH, *mmol/l*	2.6 (1.4–5.6)
Life-sustaining therapy limitations, *n (%)*	11 (27.5)
ICU mortality, *n (%)*	20 (50)

Quantitative parameters are presented as median and interquartile range (IGR, 25^th^-75^th^ percentile).

Two (5%) patients underwent a surgical procedure (in both cases after unsuccessful embolization). Active bleeding was significantly associated with embolization in univariate analysis (OR 6.88, 95% CI [1.45; 40.38], *p* = 0.01), but 4 patients were treated with embolization although they did not have active bleeding on the CT scan. Delay between embolization was not significantly associated with better or worse diagnosis.

Neither of the patients with VKA treatment received vitamin K and only 4 patients (10%) received protamine. One patient received tranexamic acid, another one received fibrinogen and a further patient received prothrombin complex concentrates.

There was no significant difference in outcome in terms of mortality according to the number of red cell concentrates, platelet or plasma transfusions.

### The prognosis of patients who develop a iatrogenic IPH in ICU is extremely severe, especially for the patients who have intravascular coagulopathy

Iatrogenic IPH was associated with hemorrhagic shock in a majority of patients: vasopressors were introduced or increased by more than 20% of the dose prior to the diagnosis in 30 (75%) cases and the median maximum dose of norepinephrine used in the 24 hours surrounding the diagnosis of IPH was 0.38 μg/kg/min. Decisions to limit life-sustaining therapy were taken in 11 (27.5%) cases (all the patients but one died subsequently). Twenty (50%) patients who developed IPH in the ICU died there. IPH was complicated by the presence of disseminated intravascular coagulopathy (defined by a JAAM 2006 score ≥ 4, (8)) in the three days following the diagnosis of IPH in 11 (27.5%) cases. Disseminated intravascular coagulopathy (see Table 5) was the only risk factor that was significantly associated with mortality in multivariate analysis (OR 6.91, 95% CI [1.28; 58.8], *p* = 0.04).

**Table 5:** Multivariate analysis of mortality risk factors of IPH in ICU.

	Total *n =* 40	Alive *n* = 20	Dead *n* = 20	OR	95% CI for OR	*p* value
**Disseminated intravascular coagulopathy, *n (%)***	**11 (27.5)**	**2 (10)**	**9 (45)**	**6.91**	**1.28–58.8**	**0.04**
Dialysis in ICU, *n (%)*	20 (50)	13 (65)	7 (35)	0.27	0.06–1.13	0.08
Body mass index	27 (23–32)	32 (26–36)	26 (23–29)	0.85	0.87–1.02	0.15

Quantitative parameters are presented as median and interquartile range (IGR, 25^th^-75^th^ percentile).

## Discussion

While the issue of IPH and retroperitoneal bleeding at admission in ICU has been discussed elsewhere [10, 11], the incidence and consequences of spontaneous iatrogenic ilio-psoas hematomas occurring within and complicating ICU stays have never been assessed yet. To our knowledge, our study is the first to assess the proportion of patients developing an IPH during their ICU stay, to identify potential risk factors and to describe both their treatment and prognosis. This study focused on spontaneous IPH rather than other hematoma (especially other retroperitoneal hematoma) that often have clearer identifiable causes.

IPH is a rare complication of ICU stay: we report an incidence of 3.8 cases/1000 patients who stayed more than 72 hours in ICU. This incidence is higher than the one reported in a recent study (3.0/1000 admissions) which considered a cohort of patients who were admitted for IPH or who developed IPH while they were hospitalized in ICU (50 and 27 patients, respectively) [10]. However, this latter study did not distinguish between the two populations in their analysis and did not address the specific problem of IPH as an adverse effect occurring in ICU. We studied patients who developed IPH only after 72 hours past their admission and calculated the incidence by including patients who stayed at least 72 hours in the ICU.

Though rare, IPH is a particularly severe complication when occurring in ICU patients. Indeed, 75% of the patients required the introduction or a significant increase of vasopressor drugs following the diagnosis of IPH, 25% of the patients in our cohort required re-intubation and 27.5% developed a disseminated intravascular coagulopathy. Twenty-six (65%) patients developed a thrombocytopenia. Moreover, half of the patients who developed an IPH died in the ICU, compared to a mortality rate of 22% during the same period for the global ICU population of patients who were hospitalized during more than 72 hours (see Fig 1). As with septic shock-induced disseminated intravascular coagulopathy [12–14]., hemorrhage-induced disseminated intravascular coagulopathy was associated with a significantly higher mortality rate in multivariate analysis (OR 6.91, 95% CI [1.28; 58.8], *p* = 0.04). The mortality rate of our cohort is higher than the one reported in the study above (30%), suggesting that IPH occurring during ICU stay may be more severe (or affect patients who are more severe) than IPH that leads to ICU admission [10]. Other studies reporting cases of IPH, that included patients who did not necessarily require admission in ICU, reported significantly lower mortality rates. One study reported a one-month mortality rate of 10% in a cohort of 89 patients who were diagnosed with IPH in the emergency room [5]. Another study that did not focus specifically on ICU reported a mortality rate of 20% [4]. An older account dating back to 1997 included 29 patients who were diagnosed with IPH and did not require admission in ICU reported only one death in its cohort [6].

Embolization was used for more than half of the patients and only 2 patients (5%) were treated surgically (both patients treated surgically had been treated by embolization first and neither survived). The use of embolization was significantly associated with active bleeding on the CT scan (OR 6.88, 95% CI [1.45; 40.38], *p* = 0.01) but we did not find any other factors significantly associated with embolization (in particular the presence of shock or the SOFA score). While the older study mentioned above [6] reported a much higher rate of surgical intervention (24 surgical procedures out of 29 cases), interventional radiology and intra-arterial embolization is the *de facto* modern gold standard treatment in spite of the lack of formal evidence-based studies supporting such practice [3]. Our study did not show any link between embolization procedures and ICU outcome. This could be explained by a lack of statistical power or by the fact that the population of patients who underwent an embolization procedure had a potentially more severe IPH. Larger observational studies could help define the place of embolization procedures for patients with IPH but, although there is no evidence-based study supporting this practice, it seems difficult, from an ethical point of view, to resort to randomized studies in order to explore this question.

Our patients were characterized by a number of salient features. First, 42.5% of them are obese (BMI>30mg/m^2^). The link between high BMI and IPH is not straightforward. In the context of spontaneous hematomas occurring in ICU, we hypothesize that the sheer stress due to the manipulation of patients by physical therapists, nurses or doctors (while weighing, cleaning, examining or transporting patients) significantly increases with body weight and might result in “traumatic” IPH. Another hypothesis is that endothelial dysfunction can be associated with obesity and comorbidities associated with obesity (high blood pressure, diabetes and hypercholesterolemia) and could lead to IPH as it has shown to lead to intracerebral hemorrhage [15]. A second feature of the patients of our cohort was the high rate of dialysis. Half of the patients who developed an IPH were under dialysis before the diagnosis was made (none of them was on chronic dialysis). Hemostatic disorders of patients with end stage renal disease and platelet dysfunction of patients on chronic dialysis have been described [16, 17] and also consequence of access site-related bleeding. To our knowledge, it has not been determined whether such dysfunctions also affect patients with acute renal failure undergoing dialysis within the context of ICU.

The last notable risk factor that was identified in our study is the high number of patients with antiplatelet therapy (37.5%) and/or anticoagulant therapy (95%). Fifteen percent of patients had a long-term double antiplatelet therapy prior to being admitted and 22.5% had a double antiplatelet therapy while they were hospitalized within the ICU. These antiplatelet treatment, however, were not associated with increased mortality. Interestingly, our report of 40 cases of IPH is the first to have systematically recorded biological results for all patients prior to the diagnosis of IPH. In particular, APTTs were measured at least daily in the 48 hours that preceded the IPH diagnosis for all our patients. Interestingly, only 4 (10%) patients had an APTT ratio > 3 (the two patients who received VKA had INRs that were < 3). APTT has been used as a standard test to monitor the effect of unfractioned heparin in patients since the 1970s, including for critically ill patients [18]. However, this test has already been shown to have theoretical and practical shortcomings to monitor the effects of unfractioned heparin [19, 20]. Our study confirms that serious adverse effects can occur in patients who are treated with unfractioned heparin and who nevertheless have APTT ratios < 3. Of note, an earlier study had already shown that ICU mortality was not associated with anticoagulant overdose among patients with extra-cerebral anticoagulant-related bleeding in ICU [11].

Finally, this study confirms that the clinical and biological clues leading to the diagnosis of IPH are largely unspecific in ICU. The most frequent sign is a loss of red blood cells > 2g/dl found in 30 (75%) patients, followed by shock in 20 (50%) patients. More specific signs are less frequent: superficial hematoma was found in 3 (7,5%) patients and positive psoas sign in 2 (5%) patients.

The limitations of this study are largely related to its retrospective nature, which nevertheless seemed most appropriate given the low incidence of this pathology.

## Conclusion

Spontaneous iatrogenic ilio-psoas hematomas are a rare, but life-threatening complication of ICU stays with a mortality reaching 50%. Age, high BMI, dialysis, anticoagulant and antiplatelet therapies are associated with this complication. Monitoring the APTT of patients with unfractioned heparin in ICU does not exclude this severe hemorrhagic complication.

## Supporting information

S1 Table Dataset. (XLSX)

## Abbreviations

APTT: Activated partial thromboplastin time
BMI: Body Mass Index
ECMO: ExtraCorporeal Membrane Oxygenation
IABCP: Intra Aortic Balloon Counter Pulsation
ICU: Intensive Care Unit
INR: International Normalized Ration
IPH: Ilio-Psoas Hematoma
JAAM: Japanese Association for Acute Medicine
SAPS II: Simplified Acute Physiology Score II
SOFA: Sequential Organ Failure Assessments (SOFA)
TAVI: Transcatheter Aortic Valve Implantation
VKA: Vitamin K Antagonist
